# A new genus and a new species of Ectrichodiinae from French Guiana and an updated key to the genera of the New World (Hemiptera, Reduviidae)

**DOI:** 10.3897/zookeys.968.54291

**Published:** 2020-09-16

**Authors:** Hélcio R. Gil-Santana, Jader Oliveira, Jean-Michel Bérenger

**Affiliations:** 1 Laboratório de Diptera, Instituto Oswaldo Cruz, Av. Brasil, 4365, 21040-360, Rio de Janeiro, RJ, Brazil Instituto Oswaldo Cruz Rio de Janeiro Brazil; 2 Laboratório de Parasitologia, Universidade Estadual Paulista “Julio de Mesquita Filho”, Faculdade de Ciências Farmacêuticas UNESP/FCFAR, Rodovia Araraquara Jaú, KM 1, 14801-902, Araraquara, SP, Brazil Universidade Estadual Paulista “Julio de Mesquita Filho” Araraquara Brazil; 3 IRD, AP-HM, SSA, Vitrome, IHU Méditerranée Infection, Aix-Marseille Université, Marseille & Laboratoire d’Entomologie du Museum National d’Histoire Naturelle, Paris, France Aix-Marseille Université Paris France

**Keywords:** *
Amazopothea
*, Heteroptera, male genitalia, Neotropics, *
Pothea
*, *
Pseudopothea
*

## Abstract

*Amazopothea guilberti***gen. nov. et sp. nov.** belonging to the subfamily Ectrichodiinae is described based on male and female specimens from French Guiana. An updated key to the New World genera of Ectrichodiinae is provided.

## Introduction

The subfamily Ectrichodiinae in the New World includes 22 genera and more than 100 described species to date (Gil-Santana et al. 2015). The latter authors provided a summary of the taxonomy of this group and a key to the genera of the New World. However, a lack of consensus exists between previous authors about the validity or recognition of some genera (e.g., Dougherty 1995; Carpintero and Maldonado 1996) and there is need of a taxonomic revision and a phylogenetic analysis of all these genera (Gil-Santana et al. 2015; Gil-Santana 2019, 2020). The new genus and new species, *Amazopothea guilberti* gen. nov. et sp. nov., described here gave us the opportunity to update the key of Ectrichodiinae genera of the New World (Gil-Santana et al. 2015).

It is the 23^rd^ genus of this subfamily. Most specimens of this new genus and species from French Guiana, males and females, were collected by the Société Entomologique Antilles-Guyane (SEAG) during a study on a protected area of this country. The specimens were among some important material caught using two kinds of interception traps, a window trap and a malaise trap (similar to those described by Lamarre et al. 2012).

## Materials and methods

**Figures 1–4.**
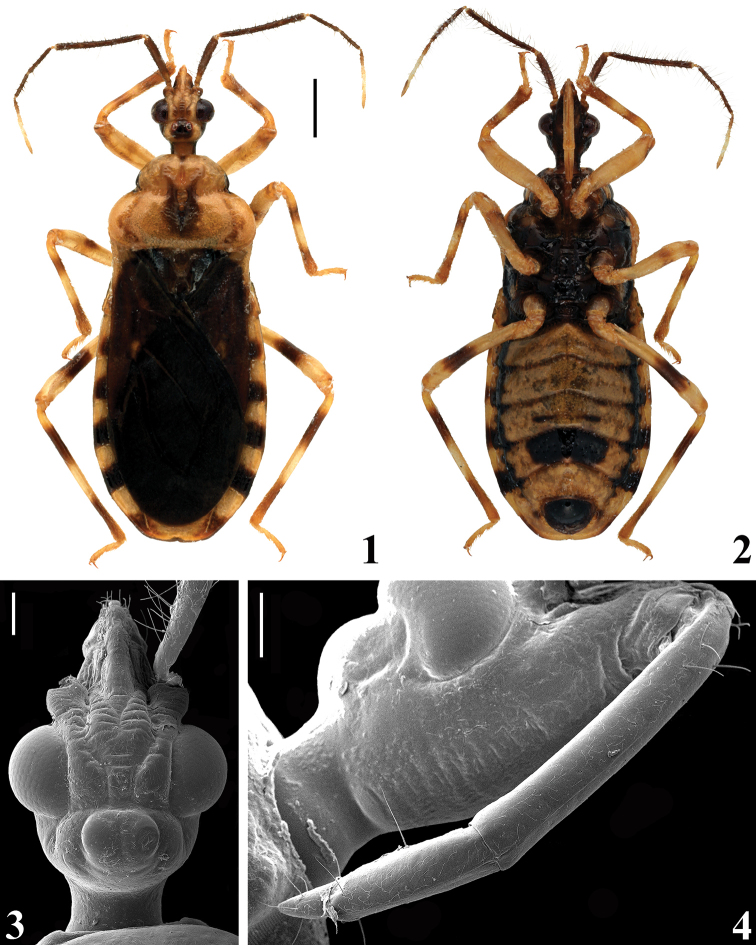
*Amazopothea guilberti* gen. nov. et sp. nov., male **1, 2** holotype: **1** dorsal view **2** ventral view **3, 4** paratype, head: **3** dorsal view **4** lateroventral view. Scale bars: 1.0 mm (**1**); 0.2 mm (**3, 4**).

Photographs of the holotype, paratypes and allotype of *Amazopothea guilberti* gen. nov. et sp. nov. (Figs 1, 2, 9, 37, 38) were taken by the third author (J-MB) using a Canon EOS Mark II digital camera with a Canon MP-E 65 mm lens. Several images were stacked using software combineZP 1.0.

The window trap used for catching insects in the forest by SEAG team is a plexiglass window with a gutter at the base, filled with a mixture of water, detergent, and salt (cf. Lamarre et al. 2012).

Scanning electron microscopy images (Figs 3–8, 10–24, 39–52) were obtained by the second author (JO). A male and a female were cleaned in an ultrasonic bath. Subsequently, the samples were dehydrated in alcohol, dried in an incubator at 45 °C for 20 min, and fixed in small aluminum cylinders with transparent glaze. Sputtering metallization was then performed on the samples for 2 min at 10 mA in an Edwards sputter coater. After this process, the samples were studied and photographed using a high-resolution field emission gun scanning electron microscope (SEM; JEOL, JSM-6610LV), as described by Rosa et al. (2010, 2014).

The specimens of *Amazopothea guilberti* examined here were very similar in size and general dimensions, with the exception of some small differences between males and females as commented below. Therefore, only one group of males (n = 11) and females (n = 4) were selected for measurements (Tables 1, 2).

**Table 1.** Measurements (mm) of male type specimens (N = 11) of *Amazopothea guilberti* sp. nov.

Measurements	Holotype	Mean	SD	range	Minimum	Maximum
Length to tip of abdomen	7.75	7.62	0.36	1.1	6.9	8.0
Head length including neck	1.56	1.53	0.09	0.25	1.37	1.62
Head width across eyes	1.0	0.97	0.05	0.13	0.87	1.0
Anteocular portion length	0.62	0.6	0.5	0.18	0.5	0.68
Postocular portion length^1^	0.31	0.35	0.03	0.06	0.31	0.37
Synthlipsis	0.5	0.48	0.03	0.07	0.43	0.5
Eye width	0.31	0.28	0.03	0.06	0.25	0.31
Fore lobe of pronotum length	0.75	0.67	0.06	0.13	0.62	0.75
Fore lobe of pronotum max. width	1.5	1.5	0.07	0.19	1.37	1.56
Hind lobe of pronotum length	0.87	0.91	0.05	0.19	0.81	1.0
Hind lobe of pronotum max. width	2.37	2.41	0.08	0.3	2.2	2.5
Abdomen maximum width^2^	2.87	2.92	0.21	0.75	2.62	3.37

^1^Excluding neck; ^2^at level of tergite V.

**Table 2.** Measurements (mm) of female type specimens (N = 4) of *Amazopothea guilberti* sp. nov.

Measurements	Allotype	Mean	SD	range	Minimum	Maximum
Length to tip of abdomen	8.4	8.48	0.3	0.7	8.1	8.8
Head length including neck	1.62	1.7	0.1	0.19	1.62	1.81
Head width across eyes	1.0	1.02	0.03	0.06	1.0	1.06
Anteocular portion length	0.56	0.7	0.09	0.19	0.56	0.75
Postocular portion length^1^	0.37	0.37	0.05	0.13	0.31	0.44
Synthlipsis	0.5	0.48	0.03	0.07	0.43	0.5
Eye width	0.25	0.25	0	0	0.25	0.25
Fore lobe of pronotum length	0.69	0.67	0.06	0.13	0.62	0.75
Fore lobe of pronotum max. width	1.62	1.69	0.05	0.13	1.62	1.75
Hind lobe of pronotum length	1.0	1.0	0	0	1.0	1.0
Hind lobe of pronotum max. width	2.5	2.48	0.08	0.19	2.37	2.56
Abdomen maximum width^2^	3.56	3.48	0.08	0.19	3.56	3.37

^1^Excluding neck; ^2^at level of tergite V.

Most of the figures of the male genitalia (Figs 27–36) and both of the abdominal segment VIII (Figs 25, 26) were produced by the first author (HRG-S). Dissections of the male genitalia were made by first removing the pygophore from the abdomen with a pair of forceps and then clearing it in 20 % NaOH solution for 24 hours. Following this procedure, the phallus was firstly recorded without inflation (Figs 32–34). The endosoma was then everted (Fig. 35) by carefully pulling on the endosoma wall, using a pair of fine forceps. The dissected structures were studied and photographed in glycerol. The microscopic preparations (Figs 29, 30, 36) were photographed using a digital camera (Sony DSC-W830). Drawings were made using a camera lucida. Images were edited using Adobe Photoshop CS6.

Observations were made using a stereoscope microscope (Zeiss Stemi) and a compound microscope (Leica CME). Measurements were made using a micrometer eyepiece. General morphological terminology mainly follows Schuh and Weirauch (2020). The (visible) segments of labium are numbered as II to IV, given that the first segment is lost or fused to the head capsule in Reduviidae (Weirauch 2008; Schuh et al. 2009). In case of terms applied particularly to the Ectrichodiinae, the terminology of general morphology follows Dougherty (1995) and Forthman et al. (2016). In general, to genitalia terms, Forthman et al. (2016) are followed.

The specimens examined will be deposited as follows: male holotype, 6 male paratypes, female allotype, and 1 female paratype in the Museum national d’Histoire naturelle (**MNHN**), Paris, France, 21 male and 2 female paratypes in the third author’s private collection (J-MB), in France. Additional paratypes will be deposited in the Entomological Collection of the Museu Nacional da Universidade Federal do Rio de Janeiro, Rio de Janeiro, Brazil (**MNRJ**), and those used to obtain SEM images will be deposited in the Dr Jose Maria Soares Barata Triatominae Collection (**CTJMSB**) of the São Paulo State University Julio de Mesquita Filho, School of Pharmaceutical Sciences, Araraquara, São Paulo, Brazil. All measurements are millimeters (mm).

## Taxonomy

### Subfamily Ectrichodiinae

#### Amazopothea gen. nov.

Taxon classificationAnimaliaHemipteraReduviidae

7F7EA06D-E5DA-521F-B364-339D23989CF8

http://zoobank.org/57A05B78-D63D-4872-9534-B8BB48FC0D11

##### Type species:

*Amazopothea guilberti* sp. nov., by present designation.

##### Diagnosis.

*Amazopothea* gen. nov. can be separated from other genera of Ectrichodiinae by the combination of characters presented in the key below. *Amazopothea* and *Pothea* Amyot & Serville, 1843 have a common characteristic which distinguishes them from all the other New World Ectrichodinae, i.e., the first (visible) labial segment elongated, longer than the second and the third (visible) together, while in the other genera this segment is shorter or at most subequal to the others together. However, *Amazopothea* can be promptly separated from *Pothea* by the presence of numerous large, rounded, deep punctations present all over sternites III–VII, while in the latter genus the integument of the sternites is generally smooth, at most with minute sparse shallow small punctations in some segments portions.

##### Description.

***Body*** integument mostly shiny. ***Head*** elongated, almost as long as pronotum (including neck); anteocular portion approximately twice longer than postocular portion (excluding neck); ratio between the total length (including neck) and maximum width across the eyes of the head around 1.6. Clypeus elongated, slightly wider at basal portion. Antennifers adjacent to the anterior margin of the eyes, their integument with moderately deep transverse subparallel sulci, more numerous and deeper on the basal half; eyes prominent, rounded in dorsal view, reniform in lateral view; transverse sulcus well marked, transverse, reaching inner posterior angle of the eye; anteriorly, a pair of longitudinal sulci running from transverse sulcus, where they are close but diverging until the level of the anterior margin of the eye, where they become slightly convergent to end near the inner side of the apex of antennifers; between them, integument presenting several parallel transverse incomplete impressions, which become more numerous and deeper anteriorly, forming sulci, similar to those on antennifers. Vertex not elevated. Ocellar tubercle prominent, large, undivided, ocelli rounded, the distance between them subequal or larger than the diameter of each ocelli; antenna inserted proximal to midpoint between anterior margin of the eyes and apex of the head; scape surpassing the apex of the head by its distal half to distal two thirds, somewhat curved and enlarged towards apex, shorter than pedicel; the latter slightly curved; flagellum slender, divided in pseudosegments, two basiflagellomeres and four distiflagellomeres; basiflagellomeres thinner than pedicel, the first basiflagellomere slightly longer than the second; distiflagellomeres somewhat thinner than basiflagellomeres, subequal in length. Labium moderately thick, segment II (first visible) straight, approximately twice longer than the segment III, also longer than the others together, by approximately 1.4 to 1.5 times, its apex approximately at level or distal to the posterior margin of the eyes; segment III somewhat thinner towards apex; segment IV, shorter, tapering, reaching stridulatory sulcus approximately at its anterior fourth. Ventral surface of head with some shallow transverse linear impressions medially. Constriction between postocular portion and neck distinct. ***Thorax***: integument shiny; collar thin; anterolateral angles rounded and small; fore lobe rounded on anterior and lateral margins, shorter and narrower than hind lobe; integument slightly wrinkled, mid-longitudinal furrow on fore lobe represented by a deep median longitudinal depression, variable in deepness and size on approximately midportion of posterior half, besides that, in some specimens, from anterior margin to the depression, a shallow and flattened sulcus is present too; at median portion of transverse furrow, a large fovea, variable in size, but always prominent, below which the mid-longitudinal furrow is represented by some large and deep punctations, progressively smaller towards posterior margin, shortly exceeding the distal half of the hind lobe or extending to the anterior portion of distal third of hind lobe; sometimes the punctations are fused to each other, resulting in larger and less numerous ones; transverse furrow distinct, carinulate, interrupted at median portion, distant from the median fovea by a distance subequal to the transverse diameter of the latter; the portions between the fovea and each transverse furrow are elevated, forming a pair of short ridges beside the central fovea. The transverse furrow continues on propleura, ending at short distance above the base of the propleural posteroventral process described below. Posterolateral furrows of pronotum distinct, their basal portion almost contiguous or somewhat distal to the transverse furrow, formed by a series of shallow punctations, which are somewhat larger, deeper and converge to the direction of scutellum base at distal portion; humeral angles rounded. Scutellum with irregular borders and a shallow, relatively small median depression; prongs widely separated at the base and parallel or subparallel towards their apices. Supracoxal lobes of propleura somewhat prominent, those of meso and metapleura not; propleura with posteroventral elongated processes, directed posteromedially, just posterior to laterodistal third of fore coxa, above lateral portion of anterior margins of mesosternum. Integument of mesopleura mostly smooth; slightly rugose on posterior third and on supracoxal lobe; integument of metapleura and of the respective supracoxal lobe rugose, with several linear subparallel irregular shallow ridges, superior margin slightly thickened and curved. Prosternum wider on approximately anterior half, moderately large, prolonged between fore coxae, apex rounded and surpassing them, reaching mesosternum, with its median portion occupied by the stridulitrum. Mesosternum anteriorly to middle coxa mostly flattened and with smooth integument; its median portion, just posterior to apex of process of prosternum, depressed on anterior margin and with some transverse sulci laterally, below which a small oval depression on midline, with elevated borders; laterally to the latter, a pair of subrectangular small depressions; middle coxae bordered by elevated margins anteriorly and medially; between them, a moderately elevated area with integument marked by few shallow transverse sulci. Metasternum short; median portion nearly squared, integument smooth, posterior margin elevated. Fore coxae close, separated by a distance somewhat longer than approximately half the width of each of them; middle and hind coxae separated from each other by a distance approximately equivalent to somewhat more than twice and approximately 1.5 times the width of each of them, respectively. Fore and middle femora subequally long, the former somewhat thickened, except at basal and distal portions and the latter, slightly thickened subapically; hind femora longer, slender, somewhat thickened subapically. On middle femora, a median ventral shallow and thin crest running from basal portion to near distal portion, imperceptible in some specimens. Tibiae straight, slightly longer than the correspondent femora; fore tibiae thicker at apex, in which the anterior margin is prominent and there is a mesal comb; mid and hind tibiae only somewhat thicker at apex; tibial pad on fore and middle tibiae very small. All tarsi slender, three-segmented. Hemelytra generally dull; moderately shiny on base of dorsal surface, laterally, and on lateral portion, basally (the same portions in which the coloration is pale yellowish). ***Abdomen***: connexivum with posterolateral angle between segments II and III somewhat prominent. Tergite I narrow; its spiracles visible dorsally somewhat far from lateral margin; anterior margin carinulate only laterally; other tergites carinulate on all extension of anterior margins. Integument of tergites II–VII and half to two-thirds of respective inner portion of dorsal connexival segments generally covered by punctations, which are larger and deeper on the tergites; those of segment VII are less prominent; outer margin of connexivum, distal margins of the tergites, more extensively in the last tergite, with smooth integument. Tergite II with its median portion somewhat lowered and bordered by longitudinal ridges. Scars of dorsal abdominal glands openings (dag) on median anterior margins of tergites V and VI, that on the latter much larger than the one on tergite V. Sternites with shiny integument; sternite II narrower than the following segments, its median portion somewhat elevated and with the integument slightly rugose; sternites II and III separated by canaliculae; other intersternite furrows more evident in median portion, as a thin line, and almost imperceptible laterally, the furrow between segments VI and VII more marked, especially in the female. Integument of sternites III–VII with numerous large, rounded, profound punctations. These punctations are distributed in two main groups in each segment: irregularly aligned below the intersternite furrows on segments IV–VII and grouped roughly as transverse irregular rows on approximately the median portion of each segment; they are absent at lateral portion of sternite III, while on sternite VII they are more randomly distributed, including the space between the proximal line of punctations and also the distal margin of the segment, portions in which, in general, there is no punctation on the other sternites. Ventral portion of connexival segments much narrower than dorsal portion; their integument entirely smooth. Male: segment VIII not visible externally, sclerotized on ventral portion, which is mostly translucent, except on darkened basal margin; the segment becomes wider towards posterior margin; both basal and distal margins curved, the former more than the latter; postero-ventral margin, narrowly elevated, except at lateral portions; dorsal portion membranous and narrower; spiracles on dorsal margin of ventral portion. Female: external genitalia with tergite X distinct.

##### Distribution.

French Guiana.

##### Etymology.

The name of the new genus was composed by the word Amazo-, from Amazon, as a tribute for this region in which this remarkable species lives and also because it holds an outstanding biodiversity that must be preserved for future generations. The second word composing the name, *Pothea*, refers to its apparent proximity of the new genus to this genus. The gender is feminine.

#### Amazopothea guilberti sp. nov.

Taxon classificationAnimaliaHemipteraReduviidae

6F540360-AD34-5D59-8E78-F611060FBE10

http://zoobank.org/F9FD080E-F93F-48BD-8BBF-F206F05D6BB6

Figures 1–4 , 5–9 , 10–15 , 16–23 , 24–31 , 32–36 , 37–40 , 41–46 , 47–52

##### Type material.

French Guiana, Holotype, male, Patawa, Montagne de Kaw, 20.xi.2001, piège malaise, J. Cerda leg. (MNHN); Allotype female, Nouragues, 20.iv.2010, piège vitre, SEAG leg. (MNHN); Paratypes: 14 males, Nouragues, 30.xi.2009, piège vitre, SEAG leg. (4/MNHN; 10/J–MB); 2 males, Montagnes des chevaux, 12.xi.2011, piège vitre, SEAG leg. (1/MNHN; 1/J–MB); 1 male, pk 37, Mt de Kaw, 16.XI.2001, malaise, J. Cerda leg. (J–MB); 1 male, Nouragues, 11.xii.2009, piège vitre, SEAG leg. (J–MB); 1 male, Montagne des chevaux, 20.xii.2008, piège vitre, SEAG leg. (J–MB); 1 male, Nouragues, Parare, 9.iv.2010, piège vitre, SEAG leg. (J–MB); 2 males, Saül, 22.iii.2011, piège vitre, SEAG leg. (1/MNHN; 1/J–MB); 2 males, Nouragues, Parare, 13.viii./2010, piège vitre, SEAG leg. (J–MB); 3 males, Trésor, 29.xi.2010, piège vitre, SEAG leg. (J–MB); 1 female, Saül, 13.viii.2010, piège vitre, SEAG leg. (J–MB); 1 female, Nouragues, 20.iv.2010, piège vitre, SEAG leg. (MNHN); 1 female, RN2, pk 65, 10.viii.2008, piège vitre, SEAG leg. (J–MB); Bélizon, vii.2001, H. Gaspard leg., 2 males (MNRJ), 1 male, 1 female (CTJMSB).

##### Description.

**Male.** Figures 1–36. Measurements are given in Table 1.

**Figures 5–9.**
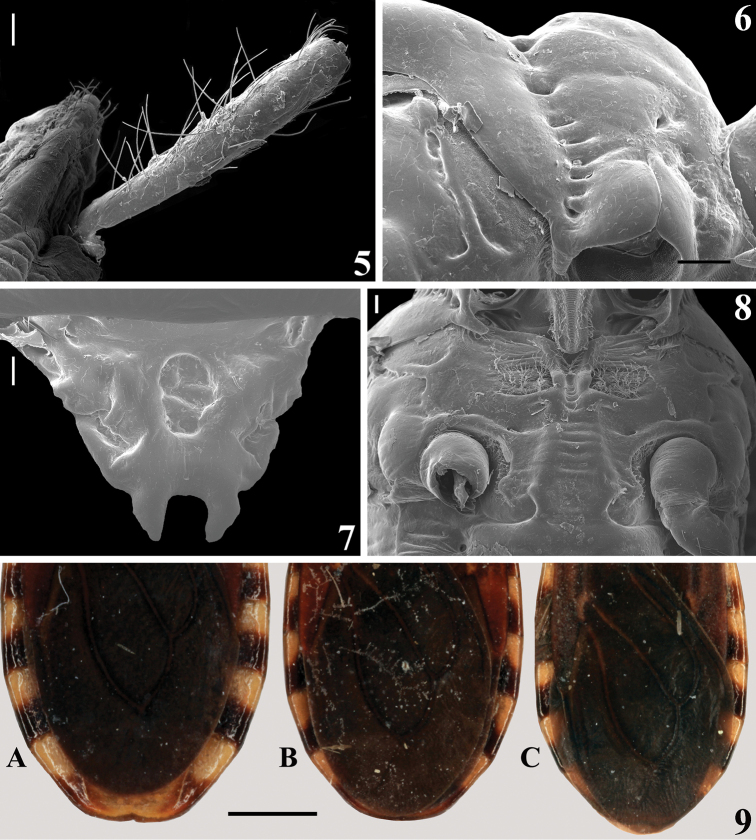
*Amazopothea guilberti* gen. nov. et sp. nov., male paratypes **5** right scape and apex of the head, dorsal view **6** pronotum, lateral view **7** scutellum, dorsal view **8** apical portion of prothorax and mesosternum, ventral view **9** approximately apical half of hemelytra and abdomen of three males, with hemelytra not reaching (**A**), reaching (**B**) or slightly surpassing (**C**) the apex of the abdomen, dorsal view. Scale bars: 1.0 mm (**9**); 0.2 mm (**6**); 0.1 mm (**5, 7, 8**).

**Figures 10–15.**
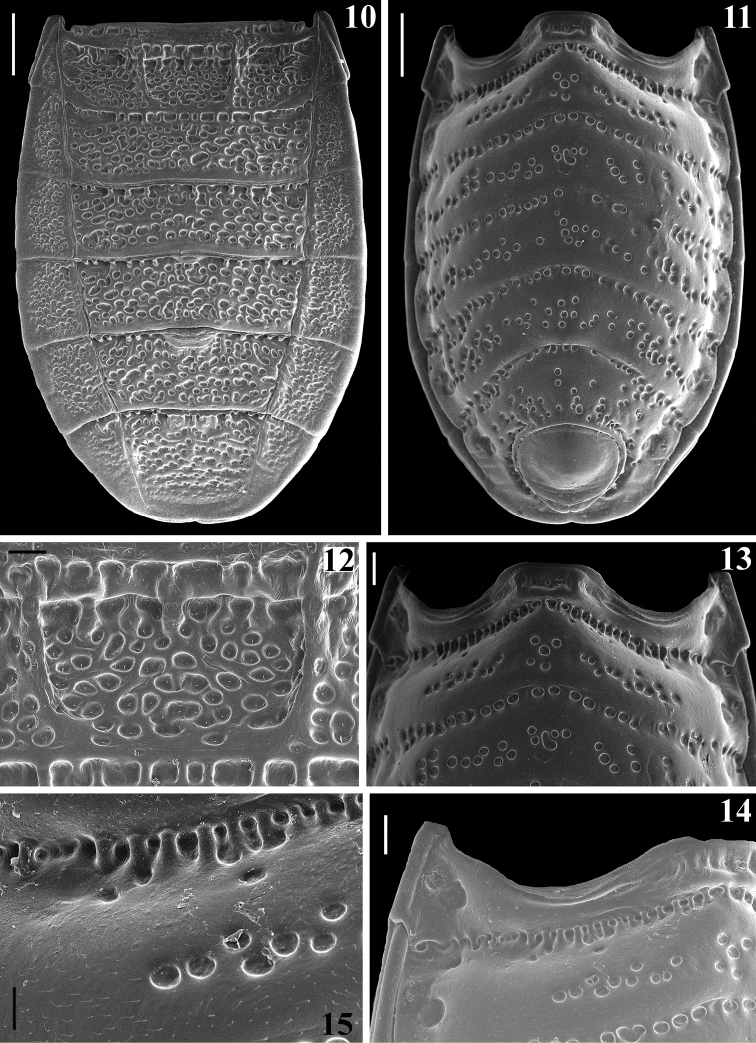
*Amazopothea guilberti* gen. nov. et sp. nov., male paratype, abdomen **10** dorsal view **11** ventral view **12** median portion of tergite II **13–15** ventral view **13** sternites II, III and IV (basal portion) **14, 15** right side of sternites II and III **14** except apico-lateral portion of sternite III **15** mediolateral portion, including canaliculae between these sternites. Scale bars: 0.5 mm (**10, 11**); 0.2 mm (**13, 14**); 0.1 mm (**12, 15**).

**Figures 16–23.**
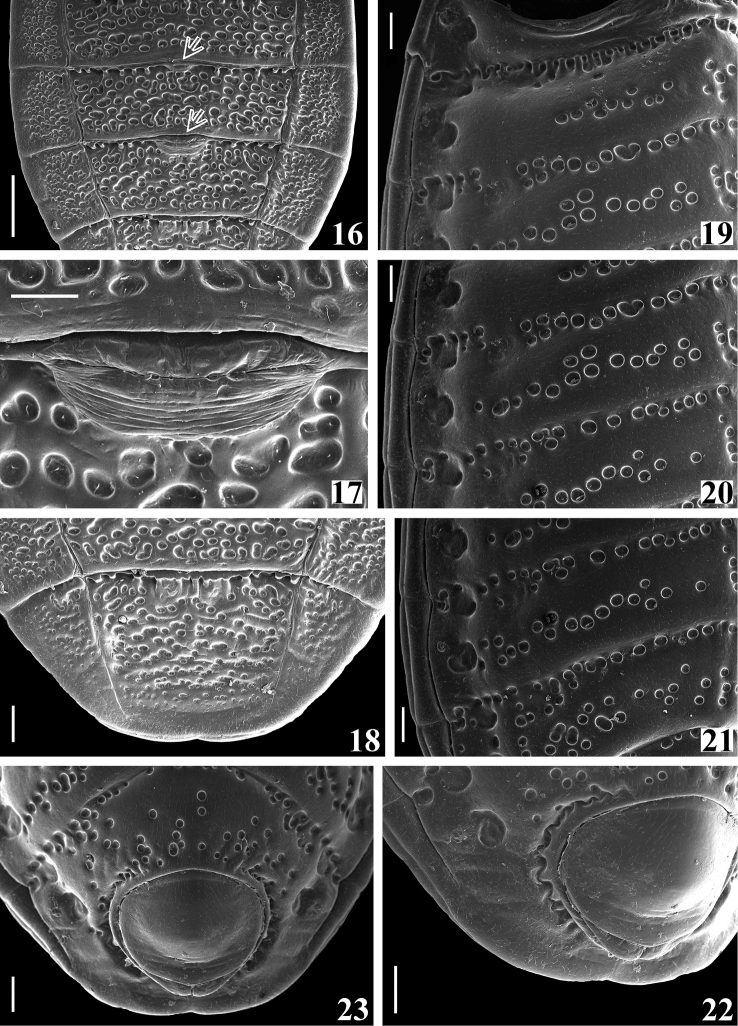
*Amazopothea guilberti* gen. nov. et sp. nov., male paratype, abdomen **16–18** dorsal view **16** segments IV (except basal portion), V–VI and VII (basal portion), arrows point to the dag on tergites V and VI **17** dag on median anterior margin of tergite VI **18** segments VI (distal portion) and VII **19–22** right side of segments, latero-ventral view **19** III and most part of II and IV **20** III (distal portion), IV and V (except latero-distal portion) **21** IV (distal portion), V and VI (except latero-distal portion) **22** distal portion of segment VII, including genital capsule **23** segment VII, including genital capsule, ventral view. Abbreviation: dag: scar of dorsal abdominal gland opening Scale bars: 0.5 mm (**16**); 0.2 mm (**18–23**); 0.1 mm (**17**).

***Coloration***: general coloration pale to pale yellowish with darkened to brownish or blackish portions or markings (Figs 1, 2). ***Head***, including neck mostly pale dorsally and darkened or blackish ventrally and laterally behind eyes (Figs 1, 2); ocellar tubercle, median portion in the space between eyes and apices of antennifers dark (Fig. 1); ventrally, on the area between eyes and base of labium with small or more extensive pale markings, which, in the latter case can cover all the area; antennal segments mostly brownish; last three distiflagellomeres yellowish (Figs 1, 2); labium mostly pale (Fig. 2), with faintly or ill-defined darkened portions such as the basal portion of the first visible segment, lateral portions of the second visible segment and distal segment. ***Thorax***: pronotum mostly pale yellowish; anterolateral angles blackish; median fovea and midlongitudinal furrow on hind lobe and the area beside it, in variable extension, darkened, brownish to pale brownish (Fig. 1), in some specimens the midportion above the median fovea, on approximately distal half of fore lobe, has the same set of coloration; scutellum darkened, its median portion, including its depression, pale (Fig. 1); its prongs sometimes paler at their apices too. Pleurae and thoracic sterna mostly blackish (Fig. 2); pro, meso and metapleura with a median yellowish spot, above supracoxal lobes, variable in size and presence among specimens; stridulitrum paler; in some specimens, basal portion of meso and metasternum pale. ***Legs*** generally pale to yellowish pale with the following darkened portions or markings (Figs 1, 2): fore and middle coxae partially or completely darkened; fore and middle femora with an ill-defined median or submedian incomplete brownish annulus and apex faintly darkened; these markings can be variable or absent; hind femur with a somewhat large submedian distal brownish to brownish black annulus and apex faintly marked or not marked; tibiae with a subbasal brownish annuli, larger on hind tibiae; faintly darkened subapically or with approximately the apical third or apical quarter darkened; tarsi yellowish. Hemelytra mostly brownish; yellowish on the base of the dorsal surface, laterally, and on the lateral portion, basally; a pale spot on the basal angle of the second discal cell of membrane, sometimes the basal portions of the veins of this cell are also pale (Fig. 1). ***Abdomen***, in dorsal view: tergites (examined in few specimens) pale brownish with some faintly darkened portions, such as intersegmental bridge between the first and the second segment, except its lateral portions, a transverse thin subapical stripe on tergite VI and a median subbasal small spot on the last tergite; connexivum clearly alternating from distal dark to basal pale whitish areas, each approximately occupying half of the respective segment (Fig. 1); in ventral view (Fig. 2): sternites mostly pale yellowish; sternite II blackish, except at lateral portions; a pair of lateral undulating black bands, which run from sternite III, contiguous with blackish coloration of the previous sternite to distal portion of sternite VI or basal portion of sternite VII, where the bands become thinner; a specimen with two pairs of transverse brownish parallel thin brownish stripes at the median portion of sternite V; sternites V and/or VII sometimes with small dark spots lateral to midline, variable in size and shape; a somewhat large median subrectangular blackish large spot on sternite VI, which in some specimens is smaller, incomplete, spotted with pale coloration at its center or divided forming a pair of submedian spots, or joining the lateral band by the darkened posterior portion of the segment. The markings on the ventral connexival segments are generally the same as those of dorsal portions, but more variable, faintly marked or partially or completely absent in some segments. Exposed portion of pygophore and parameres blackish (Fig. 2); parameres darkened, their apices paler. ***Structure***: mostly as in generic description (Figs 1–8, 10–23); minimum distance between eyes in dorsal view (synthlipsis) somewhat less than twice longer than the width of each eye. Hemelytra length variable, not reaching, reaching or slightly surpassing the apex of the abdomen (Figs 1, 9). ***Vestiture***: when recorded by SEM it is possible to clearly note that the general integument is covered by minute, thin, somewhat curved, adpressed, setae (e.g., Figs 4, 6, 15). It is noteworthy that by this method, it is possible to see that in the center of most of the large punctures of the abdomen there is one of these single minute setae implanted (e.g., Figs 12, 17). Additionally, the following features of the vestiture, also evident under the stereoscope microscope, were recorded: head: eyes and ocelli glabrous (Fig. 3); sparse curved short or somewhat longer, pale to yellowish setae on distal portion of clypeus, numerous on labrum (Figs 3–5); some longer similar setae scattered on the anterior and lateral portions of the base of the first visible labial segment; the second visible labial segment with some long, erect, setae inserted on the lateral surface of median and subapical portion; the last labial segment with few scattered erect, thin, pale setae (Fig. 4). Antennal segments, except on scape (Fig. 5), covered by short, oblique, thin setae, black on pedicel and yellowish on other segments and by numerous long, erect, stout, somewhat darkened setae, approximately twice as long as the width of the scape and thrice to four times as long as the width of pedicel and basiflagellomeres, forming a pubescence; distiflagellomeres with some longer scattered elongated oblique erect setae twice to almost thrice longer than the width of the segment, which are paler on the last three segments. ***Thorax***: posterior margin and apices of propleural posteroventral elongated processes with sparse, thin, moderately elongate setae; on prosternum, laterally to stridulitrum, a pubescence formed by thin, pale setae, which become longer towards its apex (Fig. 8); some groupings of pale thin, somewhat long setae on depressed anterior margin of mesosternum, below the apex of prosternum, and on the subrectangular small lateral depressions (Fig. 8); a patch of pale curved setae on the median wall of the middle coxal cavity (Fig. 8). ***Legs***: coxa with some stout, straight, pale, thin setae on apical margin; trochanters with curved, stout, pale, thin setae, numerous on fore trochanter, which also has at least one thinner, longer subbasal setae; scattered on middle trochanter and more numerous at its inner and apical portion; and only a few on hind trochanter; fore and middle femora with curved, stout, pale, thin setae; sparse on dorsal surface of fore femur and numerous on its ventral surface, mainly on its basal half; scattered on all surfaces of middle femur; on the latter, a ventral fringe of numerous decumbent, curved, thin, very small, pale setae inserted on the median ventral crest; in some specimens this fringe is imperceptible; hind femur glabrous. Tibiae with a mid-ventral fringe of short, straight, somewhat stouter, pale setae; scattered stout pale setae on approximately basal two thirds of the segment; the setae become more numerous with interspersed longer elements towards the apex of the segment, covering all surfaces apically. Tarsi covered with numerous yellowish and golden setae, which are longer on the ventral surface.

**Figures 24–31.**
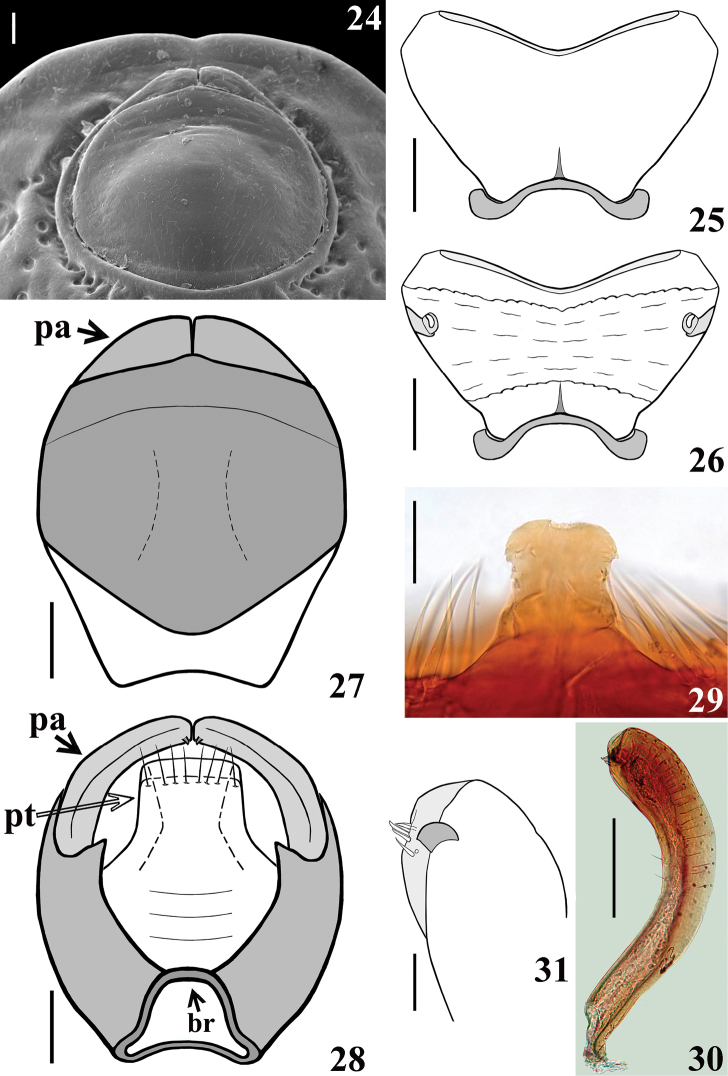
*Amazopothea guilberti* gen. nov. et sp. nov., male paratype **24** genital capsule “in situ”, ventral view **25, 26** abdominal segment VIII **25** ventral view **26** dorsal view **27, 28** pygophore and parameres **27** ventral view **28** dorsal view (br: transverse bridge; pa: paramere; pt: proctiger) **29** medial process of pygophore, anterior view **30, 31** left paramere **31** apical portion. Scale bars: 0.2 mm (**25–28, 30**); 0.1 mm (**24**); 0.05 mm (**29, 31**).

**Figures 32–36.**
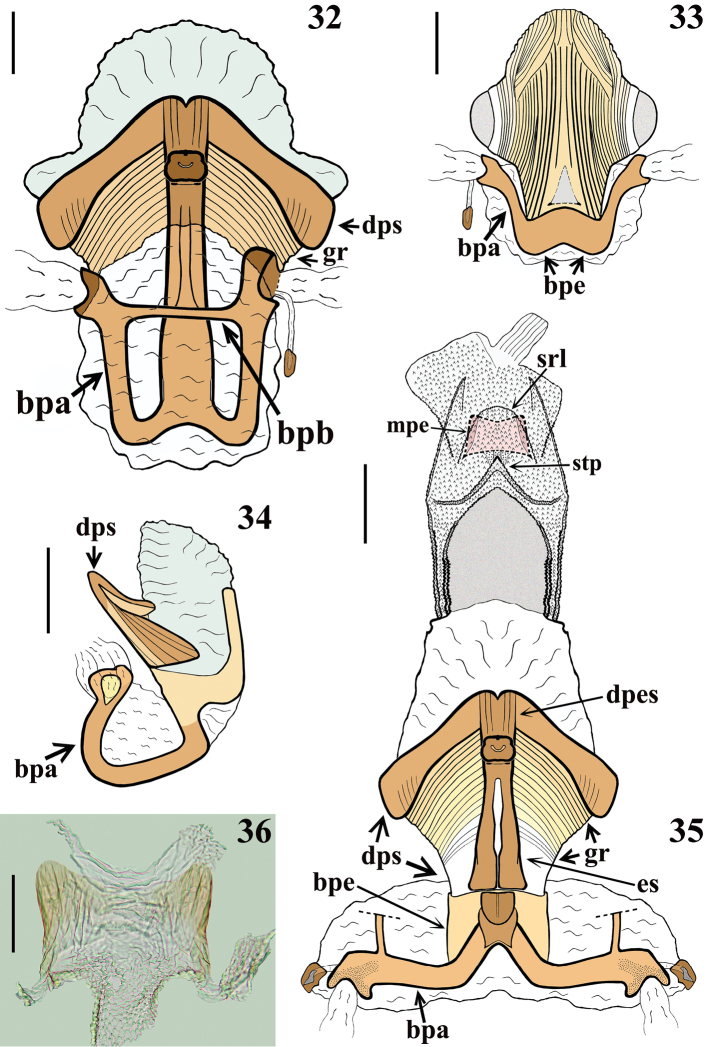
*Amazopothea guilberti* gen. nov. et sp. nov., paratype, male genitalia **32–34** phallus not inflated **32** dorsal view **33** ventral view **34** lateral view **35** phallus inflated, dorsal view. Abbreviations: **bpa**: basal plate arm; **bpb**: basal plate bridge; **bpe**: basal plate extension; **dpes**: dorsal phallothecal sclerite-endosomal struts fusion; **dps**: dorsal phallothecal sclerite; **es**: endosomal struts; **gr**: grooves; **mpe**: median process of endosoma; **srl**: flat median small rounded lobe; **stp**: subtriangular process **36** median process of endosoma, dorsal view. Scale bars: 0.2 mm (**33–35**); 0.1 mm (**32, 36**).

***Male genitalia.*** Genital capsule, in ventral and lateral views: exposed portion of pygophore sub pentagonal (Fig. 27) and rounded, respectively, integument smooth and shiny; not pigmented in the ventral non-exposed portion (Fig. 27); in dorsal view (Fig. 28): between anterior and posterior genital openings, a narrow, short moderately sclerotized dorsal (transverse) somewhat curved bridge (br); membranous areas of posterior genital opening smooth; proctiger (pt) subrectangular, posterior margin almost straight, slightly curved laterally, with a subapical row of long straight setae. Medial process of pygophore (mpp) sclerotized, subrectangular, somewhat larger towards apex; apical margin almost straight, slightly curved (Fig. 29). Parameres (pa) mildly exposed when genital capsule is in situ (Figs 22–24); their apices in contact in resting position (Figs 22–24, 27, 28). Parameres symmetrical, elongated, curved at approximately middle third; somewhat larger at apical fourth; apex truncated, with a short subapical tooth in inferior margin; mostly glabrous, with a few scattered thin setae and a group of stouter short setae medial to the subapical tooth (Figs 28, 30, 31). Phallus: articulatory apparatus with basal plate extension (bpe) much shorter than basal plate, the latter with moderately short and curved basal plate arms (bpa), connected by a narrow basal plate bridge (bpb) (Figs 32–34). Dorsal phallothecal sclerite (dps) symmetrical, enlarged to the apex, sinuous in the center of the anterior margin and somewhat sinuate laterally to the anterior margin; midlateral portions with several grooves (gr); apicolateral portions smooth, moderately thickened, and more sclerotized (Figs 32, 35). Endosomal struts (es) formed by a pair of parallel arms, nearly straight at mid portion, larger at basal portion, united at base and apex, which is continuous with the dorsal phallothecal sclerite-endosomal struts fusion (dpes) (Fig. 35). After inflation, the endosoma takes a general shape more or less tubular (Fig. 35). Endosoma wall longitudinally striated on basal portion, ventrally (Fig. 33), smooth basally, and mostly very densely minutely, spiny, with a flat median small rounded lobe (srl) located exactly dorsal to the median process of the endosoma (mpe) (Fig. 35); the latter relatively small, thin, faintly sclerotized, subrectangular, its distal margin depressed at mid portion; lateral portions finely striated (Fig. 36). A basal wide, subtriangular process (stp); in the apex of the latter, the median process (mpe) is attached by its midportion (Fig. 35).

**Figures 37–40.**
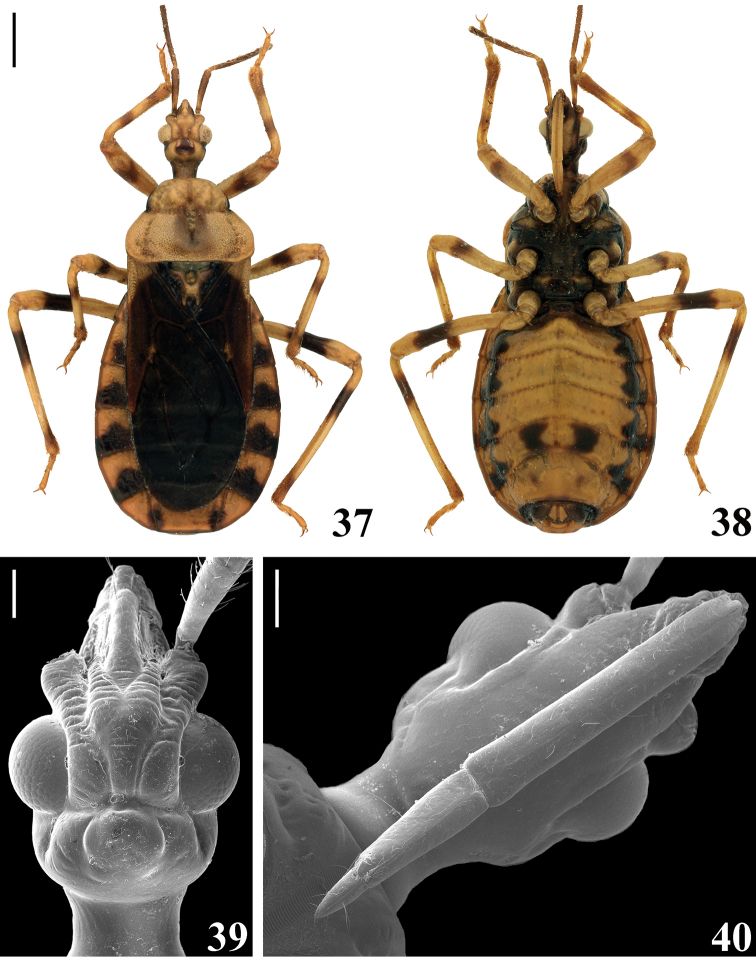
*Amazopothea guilberti* gen. nov. et sp. nov., female **37, 38** allotype **37** dorsal view **38** ventral view **39, 40** head **39** dorsal view **40** ventral view. Scale bars: 1.0 mm (**37**); 0.2 mm (**39, 40**).

**Figures 41–46.**
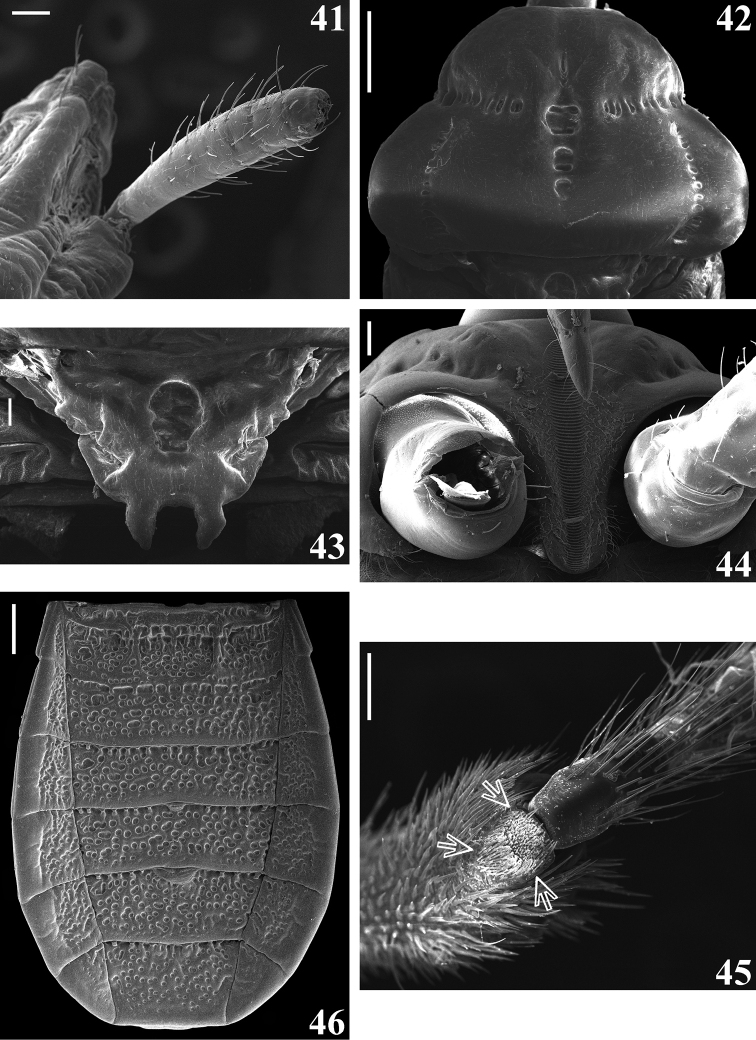
*Amazopothea guilberti* gen. nov. et sp. nov., female paratype **41–43** dorsal view **41** right scape and apex of the head **42** pronotum **43** scutellum **44, 45** ventral view **44** prosternum and fore coxa **45** middle leg, apex of tibia and basal portion of tarsus, arrows point to the tibial pad **46** abdomen, dorsal view. Scale bars: 0.5 mm (**42, 46**); 0.1 mm (**41, 43–45**).

**Figures 47–52.**
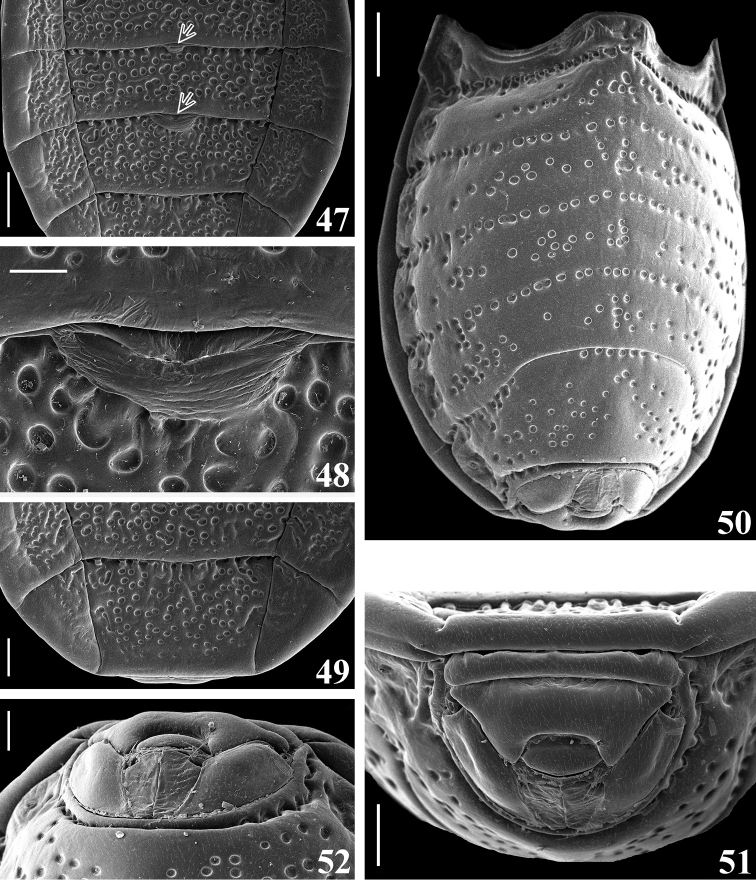
*Amazopothea guilberti* gen. nov. et sp. nov., female paratype **47–49** abdomen, dorsal view **47** segments IV (distal half), V–VI and VII (basal portion), arrows point to the dag on tergites V and VI **48** dag on median anterior margin of tergite VI. Abbreviation: dag: scar of dorsal abdominal gland opening **49** segments VI (distal portion) and VII **50** abdomen, ventrolateral view **51, 52** female genitalia **51** posterior view **52** posteroventral view. Scale bars: 0.5 mm (**47, 49, 50**); 0.2 mm (**51, 52**); 0.1 mm (**48**).

**Female.** Figures 37–52. Similar to male in general. The body was recorded as longer and the abdomen wider (measurements presented in Table 2). ***Coloration***: general coloration is the same as in males (Figs 37, 38). ***Structure*** and ***vestiture*** (Figs 39–52): head: minimum distance between eyes in dorsal view (synthlipsis) somewhat less than twice (as in males) or twice (some females) longer than the width of each eye; eyes slightly smaller than in males; antennae without pubescence (long, numerous erect setae on basal segments), scape with whitish oblique setae shorter than scape diameter, pedicel with numerous oblique pale to golden setae as long as or slightly longer than its diameter, flagellomeres with oblique pale setae approximately as long as the diameter of the respective segments; distiflagellomeres with some longer scattered elongated oblique erect setae twice to almost thrice longer than the width of the segment. Hemelytra not reaching apex of abdomen (Fig. 37). Female genitalia: posterior view of external genitalia as in Figs 50–52.

##### Distribution.

French Guiana.

##### Etymology.

The new species is named in honor to Dr Eric Guilbert (MNHN) for his significant contributions for the study of Heteroptera as well as all the help he has always given to his colleagues as the Curator of the Heteroptera Collection of MNHN.

##### Comments.

In Ectrichodiinae, the sexual dimorphism ranges from slight (e.g., body size, development of the hemelytron, and eye and ocellar size) to extreme, where females exhibit brachyptery to aptery in both pairs of wings and major modifications in other parts of the body (Forthman and Weirauch 2017). The antennae of most New World Ectrichodiinae males are pubescent on all segments with short setae becoming more abundant on the distal segments (Dougherty 1995). In females at least the first and often also the basal half of the second segment is bare or very sparsely pubescent and the distal segments with increasingly sparse elongate setae (Dougherty 1995). Some of the differences between the male and female of *Amazopothea guilberti* gen. nov. et sp. nov. examined here are in accordance with some of the sexual dimorphic features recorded in several other species of Ectrichodiinae, as follows. In male, scape, pedicel and both basiflagellomeres segments pubescent (i.e., with numerous longer, erect setae) and eyes slightly larger. On the other hand, in both sexes, the clypeus is not pointed; the legs have similar thickness (Figs 1, 2, 37, 38). No differences were observed in the coloration, while the females were mostly slightly larger than males in relation to their total length and wider abdomens (Tables 1, 2). Among the 28 males, the length of the hemelytra was different on specimens: for 20 males, the hemelytra did not reach the apex of the abdomen (Figs 1, 9A), for five, the hemelytra reached the apex of the abdomen (Fig. 9B), and for three specimens, the hemelytra slightly surpassed the apex of the abdomen (Fig. 9C). Among the four females, the hemelytra did not reach apex of abdomen (Fig. 37). Therefore, taking into account the specimens examined, *Amazopothea guilberti* gen. nov. et sp. nov. is among the Ectrichodiinae in which the sexual dimorphism is slight.

## Discussion

*Amazopothea* gen. nov. seems closer to *Pothea*, which currently includes 34 valid species (Gil-Santana 2020). Carpintero (1978) redescribed this genus, while Dougherty (1995) and Carpintero and Maldonado (1996) provided its diagnosis and Gil-Santana (2020) a summary of its taxonomic history. Both genera share many common characteristics. Among them, the presence of a first (visible) labial segment elongate, longer than the second and third (visible) together and that reaches or surpasses the posterior margin of the eye (Figs 2, 4, 38, 40), separating both of them from the other New World genera of Ectrichodiinae. However, the presence of numerous and large punctations on the sternites as recorded in *Amazopothea guilberti* (Figs 11, 13–15, 19–23, 50) is a conspicuous difference not found in any species of *Pothea*, which is why this new species deserves to be placed in a new genus, as proposed here. Few other genera of New World Ectrichodiinae have the abdominal sternites heavily punctated, namely *Cricetopareis* Breddin, 1903, *Cryptonanus* Dougherty, 1995 (only on lateral portions) and *Schuhella* Dougherty, 1995. Although in the phylogenetic proposal of Dougherty (1995) the punctated abdomen was considered as an apomorphic character, she argued that it appears to have developed independently three times. In fact, in her phylogeny these three genera are placed far from each other. As an additional possible evidence of her statement is the observation that *Daraxa carioca* Carpintero, 1980 was also described as presenting numerous punctations on the external third of the sternites which were considered similar to those recorded in species of *Cricetopareis* and absent on all the other species of *Daraxa* Stål, 1859 (Carpintero 1980). Therefore, the significance of the punctated sternites to the phylogeny of the Ectrichodiinae is in need of extensive phylogenetic studies to clarify it.

Male genitalic characters apparently have little utility in the taxonomy of Neotropical Ectrichodiinae (Dougherty 1995; Carpintero and Maldonado 1996). Dougherty (1995) posited the explanation that there were so few sclerotized structures in the phallus of Ectrichodiinae that there were no apparent differences among the various genera, with just a pair of sclerotized plates at the distal tip of the inflated endosoma. Nevertheless, differences in the male genital structures have been documented in some *Brontostoma* species (Ectrichodiinae) (Gil-Santana et al. 2005; Gil-Santana and Baena 2009; Gil-Santana et al. 2013), four species of *Pothea* (Gil-Santana 2014, 2020), *Pseudopothea paulai* Gil-Santana, 2015 (Gil-Santana 2015), and *Sinchocoris giupponii* Gil-Santana, 2019 (Gil-Santana 2019), thus highlighting the value of this character system for taxonomic and systematic studies in the subfamily Ectrichodiinae.

The male genitalia of four species of *Pothea* studied by Gil-Santana (2014, 2020) revealed several differences, mainly in the shape of the parameres, the medial process of the pygophore, the dorsal phallothecal sclerite and endosomal struts, and the median process of the endosoma, which appeared to be useful in the taxonomy of this group. The dorsal phallothecal sclerite and endosomal struts were particularly noteworthy, since their shape and “design” showed to be invariable within species and seem very particular to each species (Gil-Santana 2014).

Among the aforementioned recorded male genitalia, it is noteworthy the similarity between the grooves (gr) on the midlateral portions of the phallothecal sclerite of *Amazopothea guilberti* (Figs 32, 35) and those of *Pothea jaguaris* (Carpintero, 1980) (Gil-Santana 2014, 2020), which, however do not seem to share other particular similarities with the new species besides those common to *Pothea* species in general. On the other hand, the median process(es) of endosoma have been shown to be different for each species (e.g., Gil-Santana 2014, 2015, 2019, 2020), including now that of *Amazopothea guilberti* gen. nov. et sp. nov. (Fig. 36). Interestingly, although the lobes of the endosomal wall have been recorded in other species (e.g., Gil-Santana 2014, 2015, 2020), they are generally paired and lateral. The presence of a median lobe just dorsal to the median process of the endosoma in *Amazopothea guilberti* (Fig. 35) is striking. Only future studies of the male genitalia in other species of Ectrichodiinae, particularly those of related genera such as *Pothea*, will enable us to consider it as a unique characteristic of the new genera or species described here or eventually common to other Ectrichodiinae.

### Key to the New World genera of Ectrichodiinae based on Gil-Santana et al. (2013, 2015)

**Table:** 

1	Antennal insertion shielded laterally by a small sclerite. Scutellum with two midlateral projections and an apical blunt tip. Tarsi two-segmented. Fore and middle tibia without tibial pad	***Ectrichodiella* Fracker & Bruner, 1924**
–	Antennal insertion with at most a small process on the antennifer. Scutellum with two distal prongs. Tarsi three-segmented. Fore and middle tibia with tibial pad	**2**
2	Antennal insertion shielded laterally by a small process on the antennifer; vertex elevated, ocellar tubercle conical	***Jorgcoris* Carpintero, 1980**
–	Antennal insertion not shielded by an antennifer process; vertex not elevated, ocellar tubercle conical or rounded	**3**
3	Four antennal segments	**4**
–	Six or more [apparent] antennal segments	**5**
4	Ocelli not raised on an ocellar tubercle; abdominal sternites with heavy punctation	***Schuhella* Dougherty, 1995**
–	Ocelli raised on an ocellar tubercle; abdominal sternites without heavy punctation	***Zirta* Stål, 1859**
5	Fore femur with a ventral cleft	**6**
–	Fore femur without ventral cleft, although it may be armed on ventral surface	**9**
6	Coloration uniformly black	***Wygodzinskyocoris* Dougherty, 1995**
–	Coloration with a combination of dark and light brown	**7**
7	Abdominal sternites heavily punctated	***Cryptonannus* Dougherty, 1995**
–	Abdominal sternites lacking heavy punctation	**8**
8	Head elongate in lateral view, i.e., head length greater than head height	***Sinchocoris* Dougherty, 1995**
–	Head subtriangular in lateral view, i.e., head length and height subequal	***Doblepardocoris* Dougherty, 1995**
9	Fore femora with a row of large dentiform processes ventrally	***Borgmeierina* Wygodzinsky, 1949**
–	Fore femora unarmed or at most with a series of minute denticles or stiffened setae ventrally	**10**
10	Postocular region with a pair of blunt elevations; seven antennal segments; fore and middle femora incrassate, with a ventral carina and a row of setigerous and dentiform tubercles; length 9–9.5 mm	***Xarada* Carpintero, 1980**
–	Postocular region without a pair of elevations; seven or eight antennal segments; fore and middle femora incrassated or not incrassated, without a ventral carina and a row of setigerous and dentiform tubercles	**11**
11	Seven antennal segments; anterior pronotal lobe with a pair of paramedial carinated lobes, ocellar tubercle conical; prongs of scutellum close basally, divergent distally, spiniform	***Travassocoris* Wygodzinsky, 1947**
–	Seven or eight antennal segments; anterior pronotal lobe without a pair of paramedial carinated lobes; ocellar tubercle not conical: prongs of scutellum separated basally, subparallel.	**12**
12	Robust species of 15 to almost 40 mm in length; fore femora thickened, sometimes strongly so; middle femora less frequently thickened, both with blunt tubercles or sharp and dentiform processes set on areas with short stiff setae; fore and middle trochanters with similar armature; fore and middle tibiae slightly or strongly thickening toward apex, with tibial pad well developed	***Brontostoma* Kirkaldy, 1904**
–	Smaller and/or less robust species; femora slender or slightly thickened; different set of characters	**13**
13	Head longer than wide	**14**
–	Head length as long as or shorter than the width	**20**
14	First (visible) labial segment elongate, longer than second and third (visible) together; pronotum smooth and shiny	**15**
–	First (visible) labial segment shorter than or at most subequal to second and third (visible) together; pronotum opaque, typically rugose, seldom smooth and shiny	**16**
15	Abdominal sternites with numerous and large punctations; small species	***Amazopothea* gen. nov.**
–	Abdominal sternites lacking numerous and large punctations; small to large species	***Pothea* Amyot & Serville, 1843**
16	Second (visible) labial segment longer than first; anterior pronotal lobe with distinct sculpture, posterior pronotal lobe rugose; metasternum with two transverse carinae	***Margacoris* Carpintero, 1980**
–	Second (visible) labial segment subequal in length to first segment; different set of characters	**17**
17	First (visible) labial segment shorter than second and third together; second segment subequal to first, at most slightly longer or shorter; body red-orange and black, rarely brownish species	**18**
–	First (visible) labial almost as long as or slightly longer than second and third together; second distinctly shorter than first; body dark brown, brownish, blackish, at most with yellowish markings	**19**
18	Longitudinal sulcus of the anterior pronotal lobe well developed anteriorly, but not reaching transverse sulcus; pronotum often rugose on anterior lobe, opaque or moderately shiny; length 10–26 mm	***Rhiginia* Stål, 1859**
–	Longitudinal sulcus of the anterior pronotal lobe deep medially, but not reaching anterior or posterior margins of lobe; pronotum shiny, smooth; length 12–15 mm	***Pseudozirta* Bérenger & Gil-Santana, 2005**
19	First antennal segment approximately half as long as head; median longitudinal sulcus on anterior pronotal lobe obsolete; length 9–14.5 mm	***Pseudopothea* Wygodzinsky, 1951**
–	First antennal segment about as long as head; median longitudinal sulcus well developed on anterior pronotal lobe and extending onto posterior lobe continuously; length 8–17 mm	***Racelda* Signoret, 1863**
20	Body not flattened dorsoventrally	**21**
–	Body flattened dorsoventrally	**22**
21	With ventrolateral elevations posterior to eyes; ocellar tubercles and ocelli large to very large; legs slender, ventrally without spines or carinae; tibial pad very small, less than 1/5 length of fore and 1/10 length of middle tibiae; length 14–25 mm	***Cricetopareis* Breddin, 1903**
–	Without ventrolateral elevations posterior to eyes; ocellar callus conical or flattened; fore and middle legs strongly carinated below, femora with setigerous tubercles and dentiform spines; tibial pad on fore and middle tibiae moderately developed, extending to between 1/5 to 1/3 length of segment; body length 6–13 mm	***Daraxa* Stål, 1859**
22	Longitudinal sulcus of anterior pronotal lobe reduced to a fovea; anteocular region longer than postocular; head elongate; fore and middle femora slightly enlarged, fusiform, ventrally carinated with setigerous tubercles	***Pseudodaraxa* Carpintero, 1980**
–	Longitudinal sulcus of pronotum extending across both lobes; anteocular region much shorter than postocular; head hemispherical, vertical; fore femora enlarged basally, narrowing at apex, curved, thinly carinated ventrally, on basal 2/3, with setigerous and teeth-like tubercles; middle and hind femora similar, slender, straight, without carinae	***Pseudoracelda* Carpintero, 1980**

## Supplementary Material

XML Treatment for Amazopothea

XML Treatment for Amazopothea guilberti
